# Tannic acid-loaded zinc- and copper-doped mesoporous bioactive glass nanoparticles: Potential antioxidant nanocarriers for wound healing

**DOI:** 10.1016/j.bioactmat.2025.07.046

**Published:** 2025-08-12

**Authors:** Sara Pourshahrestani, Irem Unalan, Ehsan Zeimaran, Zhiyan Xu, Judith A. Roether, Andrea Kerpes, Christina Janko, Christoph Alexiou, Aldo R. Boccaccini

**Affiliations:** a Institute of Biomaterials, Department of Materials Science and Engineering, University of Erlangen-Nuremberg, Cauerstrasse 6, 91058 Erlangen, Germany; b Institute of Polymeric Materials, Department of Materials Science and Engineering, University of Erlangen-Nuremberg, Martensstrasse 7, 91058 Erlangen, Germany; c Department of Otorhinolaryngology, Head and Neck Surgery, Section of Experimental Oncology and Nanomedicine (SEON), Else Kröner-Fresenius-Stiftung Professorship, University Hospital Erlangen, 91054 Erlangen, Germany

**Keywords:** Mesoporous bioactive glass nanoparticles, Copper, Zinc, Tannic acid, Wound healing

## Abstract

Polyphenols such as tannic acid (TA) with antibacterial and antioxidant activities have recently attracted significant attention for wound healing applications. Mesoporous bioactive glass nanoparticles (MBGNs) have also garnered considerable interest to be employed as nanocarriers of therapeutic biomolecules. This study focuses on the fabrication of TA-loaded MBGNs which were doped with two well-known biologically active elements, copper (Cu) and zinc (Zn). The effect of TA loading on the antioxidant and biological properties of the nanoparticles was investigated in the context of potential wound healing applications. As proven with various techniques, TA was successfully loaded on CuMBGNs and ZnMBGNs. With increasing TA concentration, the phenolic content in the nanoparticles was found to increase and CuMBGNs-TA and ZnMBGNs-TA were found to possess 1,1-diphenyl-2-picrylhydrazyl (DPPH) radical scavenging activity. The nanoparticles not only showed biocompatibility towards normal human dermal fibroblast (NHDF) cells, but they were also found to be hemocompatible. In comparison to CuMBGNs-TA leachates resulting in *in vitro* wound closure rate of ∼66 %–∼83 %, the dissolution products of ZnMBGNs-TA led to higher wound closure rate (>90 %). Our results demonstrate that CuMBGNs or ZnMBGNs are suitable nanocarriers for antioxidant TA and are candidates to promote wound healing.

## Highlights

• CuMBGNs and ZnMBGNs were developed *via* a microemulsion-assisted sol-gel method.
• Biologically active TA was loaded on CuMBGNs and ZnMBGNs *via* physical adsorption.
• TA loading endowed the nanoparticles with DPPH radical scavenging activity.
• The fabricated nanoparticles exhibited hemocompatibility and biocompatibility.
• The highest *in vitro* wound closure rate was observed in the presence of ZnMBGNs-TA leachates.

## Graphical abstract

**Figure:**
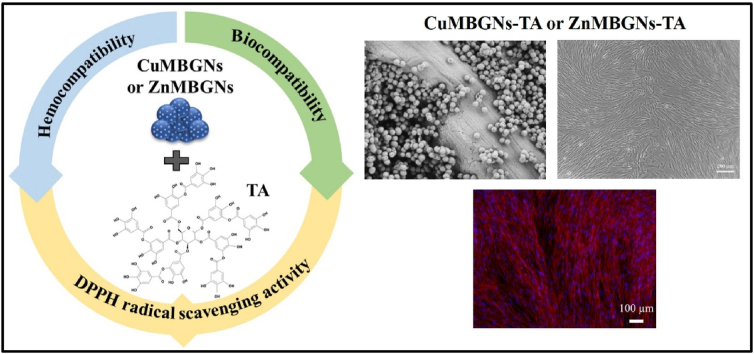

## 1 Introduction

Damage to the skin as the largest body organ can lead to serious clinical complications and can be a threat to human life [1,2]. Hemostasis is the early event starting immediately after injury, leading to blood clot formation and stopping excessive bleeding. Therefore, delays to this stage may lead to complicated consequences, or even death. During the inflammatory phase, as the second stage of wound healing, macrophages and neutrophils enter the wound site to fight off infection and protect the wound area *via* generating a high amount of reactive oxygen species (ROS) such as peroxide, hydrogen peroxide or superoxide anion as small oxygen-derived molecules. However, ROS can result in oxidative stress when they are present in high concentrations, leading to cell damage, prolonging wound healing and scar formation [[3], [4], [5]]. Thus, a balance between high or low levels of ROS is crucial. To this end, engineering multifunctional biomaterials with the ability to promote hemostasis, as well as with antioxidant and ROS-scavenging efficiencies to eradicate ROS excessive production, are of great interest for wound healing applications.

Owing to presenting attractive features, including wide availability, biocompatibility, antibacterial ability against gram-negative and gram-positive bacteria [6,7], anti-inflammatory effects [8] and, more importantly, strong antioxidant activity [9,10], tannic acid (TA) as an amphiphilic and natural polyphenol compound existing in a broad range of plants has drawn the attention of the biomaterials community [11]. TA comprising several catechol and pyrogallol moieties not only is able to interact with numerous inorganic and organic materials *via* electrostatic interactions, hydrogen bonding, and hydrophobic bonds [10,12,13] but also could interact *via* coordination bonds. TA's phenolic hydroxyl groups, for instance, can bind to metal ions such as zinc (Zn) and copper (Cu) [10,14].

Considering the aforementioned biological activities and the capability to form multiple interactions, TA has been extensively investigated to engineer diverse biomaterials for biomedical applications ranging from bone tissue engineering to wound healing [15]. Taking advantage of its antioxidant activity, capability to interact with blood proteins and accelerate blood coagulation, and its ability to inhibit bacterial infection, various TA-containing hydrogels, films, nanofibers and nanoparticles have been developed to promote hemostasis and wound healing [10]. Zheng et al. developed a hemostatic patch *via* interaction between iron ions (Fe^3+^), poly (ethylene glycol) diacrylate, TA, and N-hydroxysuccinimide-conjugated alginate [16]. The adhesive patch demonstrated hemostatic ability and good cohesive and adhesive properties, which was ascribed to the incorporated TA/Fe^3+^. In a study by Zhang et al. hydrogels with matrix system of poly (vinyl alcohol)-borax incorporated with TA and *Bletilla striata* polysaccharide were fabricated for wound healing application. TA's catechol moieties imparted tissue adhesion properties to the hydrogel. Offering more phenolic hydroxyl groups, TA also improved the antioxidant activity of the resultant hydrogels [17]. In another study by Huang et al., TA functionalization endowed a three-dimensional nanofiber sponge composed of chitosan/polyvinyl alcohol with antimicrobial and antioxidant capabilities [18]. However, to our knowledge, relatively few studies have considered the use of TA-coated nanoparticles.

Mesoporous silica (MS) nanoparticles are well known highly porous inorganic materials with great specific surface area, high pore volume, great loading capability and biocompatibility [19,20]. These nanoparticles have been loaded with TA *via* both physical absorption and chemical grafting. The effect of the nanoparticles on blood clotting was examined and TA-loaded MS nanoparticles prepared *via* physical adsorption resulted in faster clotting time compared to MS and TA-loaded MS nanoparticles fabricated *via* chemical grafting [21]. In addition to exhibiting efficient antibacterial activity, the TA-loaded MS nanoparticles were found to promote hemostasis and wound healing *in vivo* [21].

To improve the antibacterial effect and hemostatic function of MS nanoparticles, Chen et al. also prepared Janus nanoparticles *via* modification of MS nanoparticles with TA followed by embedding silver (Ag) nanoparticles through sol-gel method and redox reaction [22]. Calcium (Ca^2+^) ions were further incorporated into the system and the results showed that the resultant nanoparticles were able to accelerate hemostasis and exhibited antibacterial activity.

Whereas the antioxidant activity of TA has already been reported, studies on the effect of TA loading on the antioxidant effect of MS are still missing. Additionally, Ag nanoparticles were also reported to have a negative effect on the early stages of wound healing. For instance, Pang et al. evaluated the wound healing effect of Ag nanoparticles using a zebrafish fin regeneration model and the nanoparticles were found to impair the granulation tissue function and neutrophil recruitment was increased, thereby disrupting the wound healing process [23].

Cu as angiogenic and antibacterial element and Zn as an anti-inflammatory and antibacterial element with ability to promote different stages of wound healing are two of the most utilized therapeutic bioinorganic elements that can be embedded into various biomaterials including bioactive glasses for numerous biomedical applications, particularly bone regeneration and wound healing [24,25].

Over the past years, mesoporous bioactive glass nanoparticles (MBGNs) featuring small particle size and high textural properties (tunable pore volume and high specific surface area) close to those of pure MS materials have been utilized as nanocarriers of therapeutic ions and biologically active biomolecules, exhibiting excellent antibacterial effects, biocompatibility and proangiogenic properties [20]. Notably, numerous studies have developed MBGNs as nano powders or as fillers being incorporated in hydrogels or fibers to promote one or different stages of wound healing [[26], [27], [28]].

For instance, Paterson et al. produced Cu-containing MBGNs (Cu-MBGNs) with a composition of 85SiO_2_-13CaO-2CuO (%mol) and the results revealed that Cu-MBGNs not only exhibited biocompatibility and significant antibacterial effects against *S. aureus* and *P. aeruginosa* at concentration of 100 μg/mL, but also promoted angiogenesis at concentrations ranging from 30 to 300 μg/mL when tested *via* chick aortic ring assay [26]. The authors of this study speculated that the observed proangiogenic effect may be the result of synergy between silicon (Si) and Cu ions released from MBGNs.

A hydrogel composed of oxidized alginate (OAL) and succinyl chitosan (SCS) was made by Zhu et al., by the Schiff-base reaction followed by addition of Zn-containing MBGNs (Zn-MBGNs) and epidermal growth factor (EGF) [27]. OAL/SCS/Zn-MBGNs induced rapid wound healing and anti-inflammatory effect as compared to OAL/SCS, when it was assessed *in vivo*, confirming the positive effect of Zn-MBGNs. The hydrogel also displayed antibacterial activity, ascribed to the releasing Zn ions. The results of the study also showed that the nanoparticles and EGF were synergistically effective in enhancing collagen accumulation and angiogenesis.

In our prior research, MBGNs with different concentrations of manganese (Mn), tellurium (Te), Cu, and Zn were fabricated and their effects on wound healing were assessed *via* a series of *in vitro* assays [29]. The results showed that the effects of the nanoparticles on cell viability, antibacterial effect, reduction of blood coagulation time (activated partial thromboplastin time (aPTT) and prothrombin time (PT)), and promotion of cell migration depended on the dosages of the applied nanoparticles and concentrations of the incorporated biologically active elements [29].

By taking advantages of the great textural properties (high specific surface area, pore size and pore volume) of MBGNs and their capability to be loaded with therapeutic and biologically active ions and biomolecules, as well as anti-inflammatory and angiogenic activities of Zn [30] and Cu ions [24], respectively, and, more importantly, the antioxidant activity of TA, in this research TA-loaded CuMBGNs and ZnMBGNs were developed. In this study, we aimed to assess the effect of TA loading on physicochemical properties, antioxidant efficacy, and blood clotting capability of Cu-MBGNs and ZnMBGNs. Cell biocompatibility and cell migration were also evaluated in the presence of the leaching solution of the TA-loaded nanoparticles.

## 2 Materials and methods

### 2.1 Preparation of TA-loaded Zn- and Cu-doped MBGNs

**Fig. 1:**
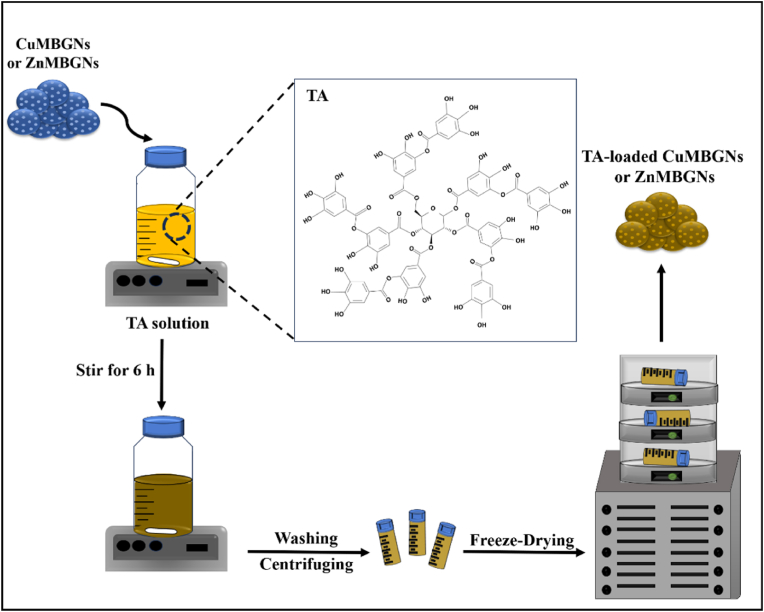
Schematic showing the preparation of TA-loaded CuMBGNs and ZnMBGNs.

CuMBGNs and ZnMBGNs in composition of 70SiO_2_-28CaO-2CuO and 70SiO_2_-28CaO-2ZnO, respectively, were prepared *via* a microemulsion-assisted sol-gel method, as reported previously [31]. Briefly, after dissolving 2.8 g of hexadecyltrimethylammonium bromide (Sigma-Aldrich) in 132 mL deionized water at 35 °C, ethyl acetate (40 mL, Sigma-Aldrich), and ammonia solution 1 M (28 mL, Sigma-Aldrich) were sequentially added at room temperature, and allowed to stir for 30 and 15 min, respectively. Afterwards, tetraethyl orthosilicate (Sigma-Aldrich), calcium nitrate tetrahydrate (VWR), zinc nitrate hexahydrate (Sigma-Aldrich) in case of ZnMBGNs and copper chloride (Sigma-Aldrich) in case of CuMBGNs were added in every 30 min interval to the solution followed by continuous stirring for 4 h. The resultant mixture was then centrifuged for 15 min and the obtained precipitates were washed with deionized water and ethanol, followed by drying at 60 °C overnight and calcination at 700 °C for 3 h. To fabricate TA-loaded CuMBGNs and TA-loaded ZnMBGNs, TA/deionized water solutions was prepared at concentrations of 3 and 5 mg/mL and then 10 mg/mL of as-prepared nanoparticles was added. The suspensions were stirred at room temperature for 6 h. Subsequently, after centrifuging and washing, the TA-loaded MBGNs were freeze-dried and then stored for further characterization (Fig. 1).

### 2.2 Physicochemical characterization of ZnMBGNs-TA and CuMBGNs-TA

The textural properties of the nanoparticles were determined *via* a porosimeter (ASAP2460, Micrometrics Instrument). The amorphous structure of the TA-loaded nanoparticles was characterized by X-ray diffraction analysis (XRD, MiniFlex 600, Rigaku). Thermogravimetric analysis (TGA, Q5000, TA Instruments) was employed to record thermal gravimetry graphs under airflow from room temperature to 600 °C. To confirm the loading of TA on Cu- or Zn-MBGNs, attenuated total reflection fourier transform infrared spectroscopy (ATR-FTIR) spectroscopy (IRAffinity-1S, Shimadzu) was utilized at 4 cm^−1^ resolution (40 scans) in the region of 400–4000 cm^−1^. The surface morphology of the TA-loaded Cu- or Zn-MBGNs was investigated by field-emission scanning electron microscopy (FE-SEM, Auriga, Carl Zeiss). The elemental composition of the nanoparticles with the highest TA loading was also assessed by energy-dispersive X-ray spectroscopy (EDS) to confirm the presence of Cu and Zn elements in the MBGNs. The average particle size of the TA-loaded samples (100 nanoparticles) was calculated using the ImageJ software (National Institutes of Health, USA).

### 2.3 Total phenolic content

Folin-Ciocalteu's (FC) (F9252, Sigma Aldrich) assay was used to estimate the amount of phenolic ring content in TA-loaded Cu- or Zn-MBGNs as described in previous studies with a minor modification [32,33]. Briefly, TA-loaded nanoparticles were immersed into methanol (34860, Sigma Aldrich) overnight at a concentration of 2 mg/2 mL, followed by addition of FC's phenol reagent solution (2 mL). After 5 min incubation at room temperature and in dark conditions, sodium carbonate (27771.290, VWR) 7.5 %w/v (4 mL) was added to stop the reaction, followed by incubation of the solution at room temperature for 90 min. UV–Vis spectroscopy (Specord40, Analytic Jena, Germany) was used to measure the amount of phenolic content of TA-loaded nanoparticles using gallic acid (GA, 27645, Sigma Aldrich) calibration curve at 765 nm. The total phenolic content was calculated in 8 mL of the mixture. Deionized water and GA were used as blank and standard phenolic compound, respectively.

### 2.4 Tannic acid release study

The release behavior of TA was assessed *via* immersing TA-loaded Cu- or Zn-MBGNs (2 mg) into 10 mL of Dulbecco's phosphate-buffered saline (DPBS, 10010023, Gibco Life Technologies, ThermoFisher Scientific) solution followed by incubation at 37 °C in an orbital shaker (80 rpm) over 28 days. At specified time points, 500 μL of PBS was removed and replaced with 500 μL of fresh DPBS. The concentration of released TA was then calculated using a UV–Vis spectrophotometer at 280 nm according to the TA calibration curve. The cumulative release of TA was calculated in 10 mL of DPBS.

### 2.5 Antioxidant activity

The 1,1-diphenyl-2-picrylhydrazyl (DPPH, D9132, Sigma Aldrich) radical scavenging assay was conducted to investigate the antioxidant properties of TA-loaded nanoparticles, as described previously [33]. Briefly, 2 mg of the nanoparticles was incubated with 2 mL methanol overnight. Then, 2.5 mL of DPPH solution in methanol (0.04 mg/mL) was prepared and mixed with the supernatant solution of methanol-TA loaded nanoparticles (0.5 mL). After 1.5 h incubation, the absorbance of the solutions was measured using UV–Vis spectroscopy at 517 nm. DPPH radical solution and methanol were used as the control and blank, respectively. The following equation was used to determine the DPPH radical scavenging activity:

DPPHradicalscavengingactivity(%)=((Ab.control−Ab.sample)/Ab.control)×100.

### 2.6 *In vitro* hemostatic response

Blood was collected from human healthy volunteers for the following experiments and the use of human blood was approved by the ethics committee of the University of Erlangen–Nuremberg (# 21-383-2-B). Experiments were conducted with blood from 3 donors.

#### 2.6.1 aPTT and PT

PT and aPTT were determined using platelet-poor plasma (PPP) to assess the effect of TA-loaded CuMBGNs or ZnMBGNs on the blood coagulation cascade. The PPP was prepared *via* centrifuging sodium citrate anticoagulated blood at 2500×*g* for 15 min. Then, 10 mg of the TA-loaded CuMBGNs or ZnMBGNs was dispersed in 100 μL PPP, followed by incubation for 15 min at 37 °C. The suspensions were further diluted with PPP resulting in final concentrations of 1 mg/mL. To determine the aPTT, after adding PPP with the nanoparticles (100 μL) into prewarmed tubes, aPTT reagent (100 μL, DiaSys, Flacht, Germany)) was added followed by addition of 25 mM CaCl_2_ solution (100 μL, Carl Roth GmbH & Co. KG, Germany). To measure PT, 100 μL PPP was used to dilute the MRX PT buffer (600 μl, DiaSys, Flacht, Germany). Afterwards, 20 μL of this mixture was added to 180 μl of PPP with the nanoparticles, followed by addition of MRX PT Owren (200 μL, DiaSys, Flacht, Germany). The coagulation times were measured using MC4 Plus coagulometer (Merlin Medical, Lemgo, Germany).

#### 2.6.2 Hemolysis assay

Lithium heparin anticoagulated whole blood (400 μL) was added to 10 mL PBS containing different amounts of the TA-loaded Cu- or Zn-MBGNs (10, 20 and 50 mg) and the mixtures were incubated for 1 h at 37 °C. After centrifuging the mixture at 800 rpm for 10 min, the absorbance of the supernatants was recorded at 541 nm, measured using Spectramax iD3 plate reader (Molecular Devices, USA). 1 % Triton X-100 solution (Carl Roth GmbH & Co. KG, Germany) and PBS mixed with blood suspension were used as positive (PC) and negative control (NC), respectively. The following formula was used to calculate the hemolysis ratio:

$$\text{Hemolysis}\;\text{ratio}\;\left(\%\right)=\left(\left({\text{OD}}_{\text{sample}}–{\text{OD}}_{\text{NC}}\right)/\left({\text{OD}}_{\text{PC}}–{\text{OD}}_{\text{NC}}\right)\right)\times 100\text{.}$$

### 2.7 Biocompatibility with NHDF cells

The *in vitro* biocompatibility of the TA-loaded Cu- or Zn-MBGNs towards normal human dermal fibroblast cells (NHDF, C-12302, Promo Cell) was assessed by the cell counting kit-8 (Sigma Aldrich, Germany). The cells were seeded in 24 well-places (at a density of 5 × 10^4^ cells/well), followed by incubation at 37 °C in 5 % CO_2_ for 24 h. Simultaneously, after sterilizing the TA-loaded CuMBGNs or ZnMBGNs by UV irradiation for 1 h, the nanoparticles at concentration of 1 mg/mL, 2 mg/mL, and 5 mg/mL were incubated in a high glucose DMEM (10313021, Gibco Life Technologies, ThermoFisher Scientific, supplemented with 1 % penicillin/streptomycin and 10 % fetal bovine serum) at 37 °C in 5 % CO_2_ for 24 h. The extracts of the nanoparticles were then obtained *via* centrifuging the mixture at 4200 rpm after 24 h of incubation. Afterwards, the DMEM was replaced by 1 mL of the extracts of the TA-loaded Cu- or Zn-MBGNs, followed by further 48 h incubation. 2 % (v/v) water-soluble tetrazolium salt (WST-8) in DMEM and the cells treated with DMEM were used as blank and control (CNT), respectively. After this time point, the extracts were removed and the cells were treated with 2 % (v/v) WST-8 in DMEM for 3 h. A microplate reader (PHOmo, Anthos Microsystem GmbH, Germany) was used to determine the absorbance at 450 nm, followed by calculation of the cell viability *via* the following equation:

Cellviability(%):((Ab.sample−Ab.blank)/(Ab.control−Ab.blank))×100.

To observe NHDF cell morphology after treatment with the extracts of TA-loaded nanoparticles for 48 h, fluorescence microscopy was utilized. In this regard, the cells were stained with DAPI, Calcein AM and rhodamine-phalloidin.

### 2.8 NHDF cell migration

The wound scratch assay was conducted to determine NHDF cell migration. NHDF cells (50,000 cells/well) were seeded into 24-well plates and incubated at 37 °C until formation of a confluent monolayer. 100 μL pipette tip was then used to make a scratch through the NHDF cell monolayers. In parallel, nanoparticle extracts were prepared *via* incubation of the TA-loaded Cu- or Zn-MBGNs (1 mg/mL) in DMEM for 24 h, followed by centrifugation at 4200 rpm. Subsequently, after washing the cells with DPBS, the cells were treated with the nanoparticle extracts. The cells treated with the nanoparticle-free DMEM were used as CNT. At appropriate time points (0 and 24 h), the scratch areas were imaged *via* light microscopy (Carl-Zeiss, Germany) and were analyzed using ImageJ. The following formula was utilized to calculate the NHDF cell migration rate over time:

$$\text{Migration}\;\text{rate}\;\left(\%\right)=\left(\left(A_{0}\;–\;A_{t}\right)/\;A_{0}\right)\times 100$$

where the initial wound area and the wound area at 24 h are specified by A_0_ and A_t_, respectively.

### 2.9 Statistical analysis

The statistical differences between groups were evaluated using one-way analysis of variance (ANOVA) with Tukey's post-hoc analysis. The data are stated as mean ± standard deviation (SD). The significance level was considered as p < 0.05 and marked by (∗).

## 3 Results and discussion

### 3.1 Characterization of ZnMBGNs-TA and CuMBGNs-TA

**Fig. 2:**
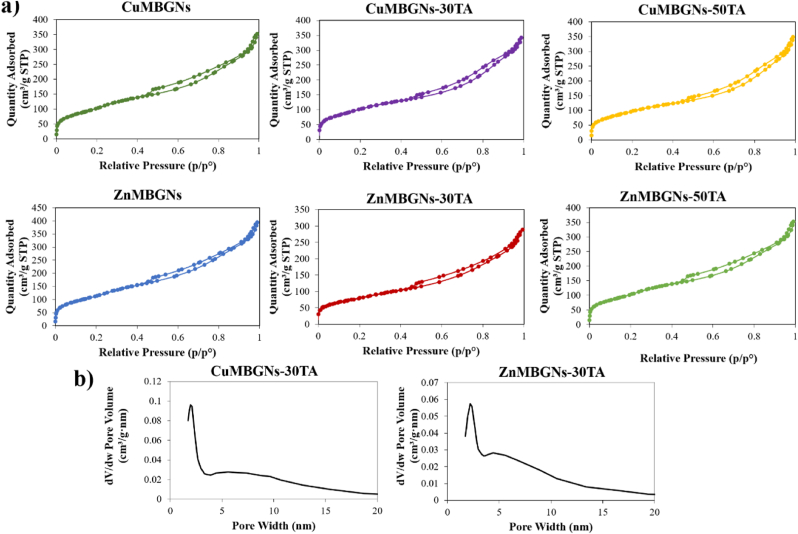
a) N_2_ adsorption-desorption isotherms of TA-loaded and unloaded CuMBGNs and ZnMBGNs. b) Pore size distribution curves of CuMBGNs-30TA and ZnMBGNs-30TA.

Textural properties of TA-loaded and unloaded nanoparticles were assessed *via* N_2_ sorption porosimetry measurements, as summarized in Table 1. As the results demonstrated, after being loaded with TA, both BET surface area and pore volume of the ZnMBGNs and CuMBGNs were reduced, and ZnMBGNs experienced a reduction of the BET surface area (293 m^2^ g^−1^) when they were loaded with 3 mg/mL of TA (ZnMBGNs-30TA). The reduction in textural properties may be ascribed to the efficient adsorption of the TA molecules inside the mesopores. The N_2_ adsorption-desorption isotherms of the nanoparticles before and after being loaded with TA are shown in Fig. 2a. Based on the IUPAC classification, all TA-loaded and unloaded nanoparticles exhibited type IV isotherms, demonstrating the presence of mesoporous structures. Fig. 2b shows the representative pore size distribution curves of CuMBGNs-30TA and ZnMBGNs-30TA, which exhibit a narrow pore size distribution centered at around 2 nm and a wider distribution for pores >4 nm.

**Table 1:** BET surface area (m^2^ g^−1^) and total pore volume (cm^3^ g^−1^) of CuMBGNs, ZnMBGNs and TA-loaded nanoparticles.

	CuO and ZnO (mol%)	TA concentration	BET Surface Area (m^2^ g^−1^)	Total Pore Volume (cm^3^ g^−1^)
**CuMBGNs**	2 mol % CuO	–	413	0.54
**CuMBGNs-30TA**	2 mol % CuO	3 mg/mL	380	0.51
**CuMBGNs-50TA**	2 mol % CuO	5 mg/mL	359	0.52
**ZnMBGNs**	2 mol % ZnO	–	449	0.60
**ZnMBGNs-30TA**	2 mol % ZnO	3 mg/mL	293	0.43
**ZnMBGNs-50TA**	2 mol % ZnO	5 mg/mL	408	0.39

**Fig. 3:**
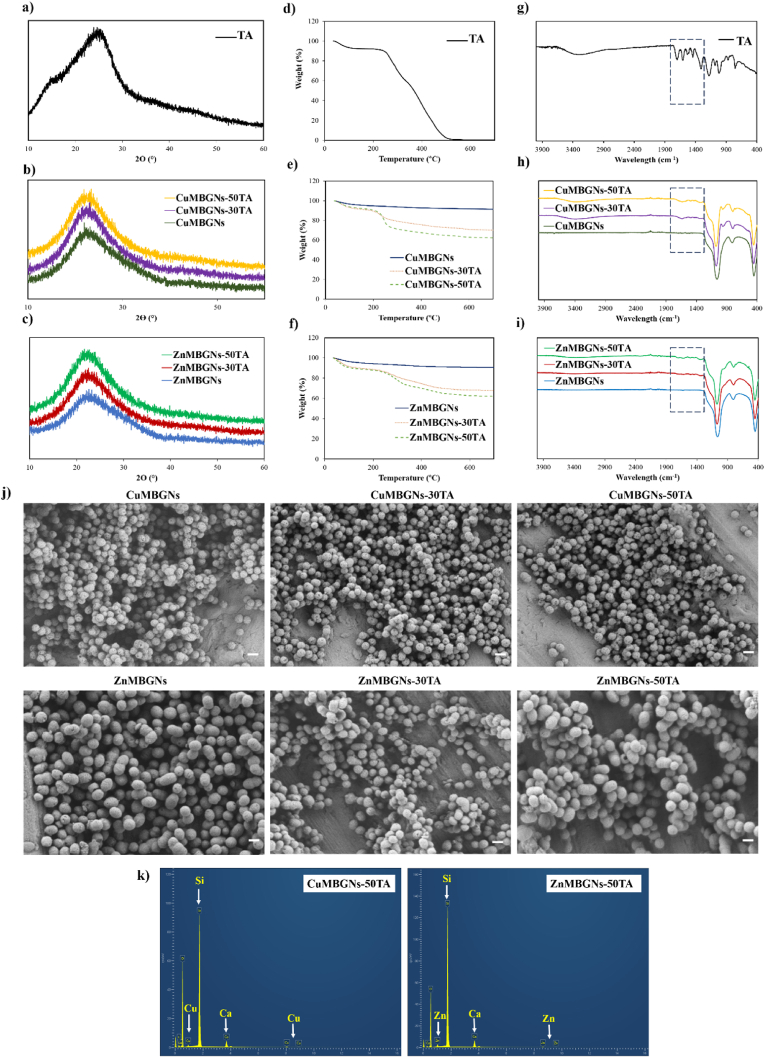
a-c) XRD patterns; d-f) TGA curves and g-i) FTIR spectra of TA-loaded and unloaded nanoparticles. j) FESEM images of ZnMBGNs and CuMBGNs before and after loading with TA (Scale bar: 200 nm). k) EDX analysis of ZnMBGNs-50TA and CuMBGNs-50TA.

To assess the structure changes of the nanoparticles after loading with TA, XRD analysis was performed (Fig. 3a–c). The XRD pattern of TA reveals a broad band at 2θ = ∼20–30°, demonstrating the amorphous nature of this compound. All the unloaded and TA-loaded nanoparticles also displayed similar patterns. A broad band was observed in the range 20°–30° (2θ), signifying the amorphous nature of the nanoparticles. These results demonstrated that the structure of the MBGNs did not change during the physical adsorption of TA.

To confirm the TA loading and assess the amount of TA, TGA was performed. As the results demonstrated (Fig. 3d–f), about 8 % weight loss from TA was observed below 150 °C in air, which can be ascribed to the loss of water. TGA results also showed the major degradation of TA occurring at about 240 °C and that TA was almost burned out at around 530 °C. The heat treatment of both unloaded ZnMBGNs and CuMBGNs up to 800 °C resulted in weight loss of around 10 % which can be related to the loss of adsorbed water and removal of residual CTAB. The TGA results of TA-loaded ZnMBGNs and CuMBGNs also showed that with increasing the amount of TA loading from 3 mg/mL to 5 mg/mL, the total weight loss of the TA-loaded nanoparticles also increased. Both CuMBGNs-50TA and ZnMBGNs-50TA with the highest amount of TA loading showed a similar total weight loss of around 38 wt %, and ZnMBGNs-30TA demonstrated a higher weight loss (of around 32 wt %) as compared to CuMBGNs-30TA (around 30 wt %). While for ZnMBGNs-50TA, a 12 wt % weight loss was observed at temperatures less than 150 °C, CuMBGNs-50TA recorded a weight loss of around 8 wt % in the same temperature range, which may be attributed to the higher ability of ZnMBGNs-50TA to adsorb water. In the temperature range of 240–700 °C, TA loaded nanoparticles additionally showed another stage of weight loss, which can be ascribed to the TA thermal degradation in the ZnMBGNs and CuMBGNs.

Fig. 3g–i depicts the FTIR spectra of pure TA and TA-loaded ZnMBGNs and CuMBGNs. A broad adsorption band appeared in the region of 2800–3700 cm^−1^ in the TA spectrum, which was assigned to the hydroxyl group (OH stretching vibration). The peaks of the ester groups at ∼1030 cm^−1^ (C–O asymmetric stretching vibration), ∼1320 cm^−1^ (C–O symmetric stretching vibration) and ∼1710 cm^−1^ (C═O stretching vibration) were also present. The spectrum also demonstrated peaks at ∼1600, ∼1514, and ∼1452 cm^−1^, ascribed to aromatic C–C stretches and at about ∼1197 cm ^−1^, which was attributed to phenolic C–O stretching [21]. Unloaded ZnMBGNs and CuMBGNs also exhibited FTIR patterns similar to that of silicate glasses. Three bands appeared at ∼400 - ∼1100 cm^−1^ (Si–O–Si rocking vibration, Si–O–Si symmetric stretching vibration, asymmetric stretching vibration)*,* indicating the formation of a silica network [34]. In the case of TA-loaded ZnMBGNs and TA-loaded CuMBGNs, in addition to the characteristic bands ascribed to the silica network, additional peaks also appeared at the regions of ∼1300–∼1700 cm^−1^, corresponding to the TA presence, confirming the successful physical adsorption of TA onto the nanoparticles, as it was also confirmed by the results of TGA.

FESEM images of ZnMBGNs and CuMBGNs before and after loading with TA are also shown in Fig. 3j. The nanoparticles exhibit near spherical (ovoidal) shape and the particle sizes fall within the range of ∼ 100–250 nm. The results illustrated that shape and size of the nanoparticles before and after loading with TA were almost similar, proving that the morphological characteristics of ZnMBGNs and CuMBGNs have not been significantly altered with TA loading. The possible reason behind the insignificant change in morphological characteristics of ZnMBGNs and CuMBGNs after TA loading may be their preparation method, which was *via* physical adsorption. This can result in a thin layer of loading without changing the particle size significantly so that a change of size cannot be clearly recognized using SEM. In a study by Liu et al., TA-AgNPs were prepared and no obvious difference was observed between the sizes of the AgNPs and TA-AgNPs based on the TEM images [35]. In another study by Sui et al., MBGNs were coated with polydopamine (PDA) followed by loading with Cu and cobalt (Co). It was speculated that the PDA coating thickness was relatively thin in comparison to MBGNs dimensions, therefore the MBGNs size did not significantly changed by the PDA coating (in the range of nanometers) [36]. In a study by Dereje et al., squaraine dyes-loaded mesoporous silica nanoparticles (Br-SQ-loaded MSNs) were prepared and the shape and size of Br-SQ-loaded MSNs were found to be similar to those of MSNs and, based on SEM/TEM images, the morphological structures of MSNs did not significantly change with the incorporation of SQ [37].

Fig. 3k shows the representative EDX spectra of the ZnMBGNs-50TA and CuMBGNs-50TA which not only confirms the presence of Si and Ca but also the successful incorporation of Zn and Cu in the MBGNs.

### 3.2 *In vitro* TA release

**Fig. 4:**
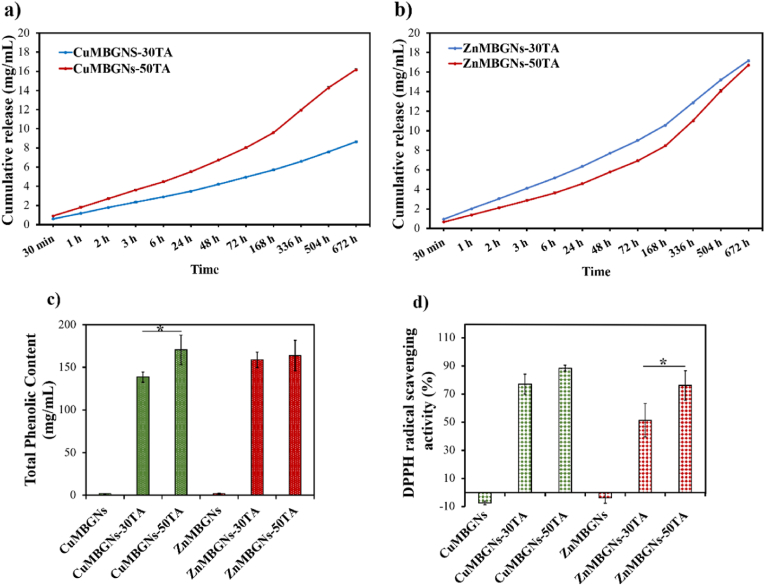
The cumulative TA release profile from a) TA-loaded Cu-MBGNs and b) TA-loaded ZnMBGNs in DPBS. (c) Total phenolic content and (d) DPPH radical scavenging activity of different nanoparticles investigated. Data were analyzed using one-way ANOVA (Tukey Test). Error bars show the mean ± standard deviation (samples in triplicates and four replicates). The symbol (∗) demonstrates the significant differences between two samples of CuMBGNs-30TA and CuMBGNs-50TA or ZnMBGNs-30TA and ZnMBGNs-50TA at p < 0.05.

The prolonged and sustained release of polyphenols may be advantageous to promote wound healing. Therefore, to assess the TA release from TA-loaded CuMBGNs and ZnMBGNs, the nanoparticles were immersed in DPBS and the TA release profile was assessed over 672 h incubation time at 37 °C (Fig. 4). Within the first 6 h, a high concentration of TA was released from the nanoparticles. The mechanism behind the TA release could be ascribed to the mesoporous channel disruption and therefore releasing TA molecules which are physically adsorbed and are present on the external surface of the mesopores or inside the pores. In comparison to ZnMBGNs-50TA (Fig. 4b), CuMBGNs-50TA (Fig. 4a) resulted in higher TA release within the first 6 h. However, the results demonstrated a continuous release of TA, observed up to 672 h for all nanoparticles, which could favor the wound healing process. In a similar study conducted by Wang et al.*,* incubation of the TA-loaded MS nanoparticles in PBS, however, led to almost 100 % TA release within 2 h [21]. As can be seen from our results, TA release was influenced by the composition of the MBGNs. While with increasing the concentration of TA loading in CuMBGNs the release of TA was increased, the amount of TA released from ZnMBGNs-TA in PBS was found to be independent of the loading amount. ZnMBGNs-30TA demonstrated a higher release of TA from ZnMBGNs as compared to that of ZnMBGNs-50TA within 336 h, followed by similar release profile after 672 h. It may be speculated that TA molecules were mostly physically adsorbed on the external surface of both ZnMBGNs-30TA and ZnMBGNs-50TA rather than adsorbed inside the mesopores and therefore a similar release profile of TA can be observed for them after 672 h incubation in PBS. In case of CuMBGNs, however, as compared to the CuMBGNs-30TA, CuMBGNs-50TA resulted in a higher release of TA. It may be proposed that in CuMBGNs-TA samples, TA not only was physically adsorbed on the external surface of the nanoparticles, but also it was entrapped inside the mesopores *via* interaction and complexation with Cu, which can delay the release of TA. It has been reported that TA can complex with Cu *via* phenolic hydroxyl groups [38]. While this possible interaction can lead to more efficient TA loading, the release behavior of TA can be influenced and its release may be hampered. It was also reported that the TA:Cu ratio is important, as increasing the loading of Cu may result in the degradation of organometallic complexes, followed by an increased concentration of Cu that was previously sequestered [39]. In our study, with increasing TA concentration, the interaction between TA and Cu may be limited, and most of the TA is likely adsorbed on the external surface of the nanoparticles. Therefore, increasing the concentration of TA resulted in a higher release of TA. In a study by Sánchez-Salcedo et al., MBGNs in the SiO_2_–CaO–P_2_O_5_ system were incorporated with 2.5 % or 4 % ZnO, followed by loading with curcumin. It was found that the curcumin release from MBGNs was faster than from nanoparticles doped with Zn. It was reported that the amphoteric oxide ZnO, formed by Zn, has the ability to react with both bases and acids. Thereby, as a result of the interaction between curcumin and Zn, the release of the curcumin was hindered in the MBGNs incorporating Zn [40]. In another study by Damian-Buda et al., MBGNs with composition SiO_2_–P_2_O_5_-CaO-Na_2_O and incorporating MgO and/or ZnO as well as B_2_O_3_ were prepared and loaded with GA. The authors reported that divalent cations such as Zn^2+^, Mg^2+^ and Ca^2+^ can be chelated by the negatively charged carboxyl group of GA, leading to a slower release of GA [41]. Overall, the TA release profile of TA-loaded CuMBGNs and ZnMBGNs indicated that TA can be released in a sustained manner, confirming the advantages of CuMBGNs and ZnMBGNs as promising nanocarriers for TA.

### 3.3 Total phenolic content and *in vitro* antioxidant analysis

The wound healing process can be regulated by ROS *via* influencing inflammation, proliferative and granulation phases, angiogenesis, and extracellular matrix formation [42]. While ROS increase in moderate level can be advantageous in the wounded area to kill bacteria, the ROS overproduction can result in damage to cells and tissues, chronic inflammation and may impede the wound healing process [42,43]. Antioxidants play a vital role to protect cells from the toxic effect of free radicals and ROS *via* neutralizing free radicals or diminishing their toxic effects *via* donating an electron, thereby preventing disease [44]. Therefore, engineering multifunctional materials with the ability to promote the wound healing process and to exert antioxidant properties is of great interest. Owing to the capability of being free radical and active oxygen species scavengers, polyphenolic compounds including TA are recognized as excellent antioxidants and have attracted worldwide attention in the field of wound healing [10,45]. It is worthy to note that phenolic OH groups are reported to promote free radical scavenging [46]. Herein, the total phenolic content of the TA-loaded ZnMBGNs and CuMBGNs was therefore determined using FC assay and GA calibration curve. As the results demonstrated (Fig. 4c), the phenolic content in methanol extracts of the TA-loaded nanoparticles increased with increasing TA concentration. Notably, ZnMBGNs-50TA and CuMBGNs-50TA were found to have higher phenolic content, as compared to other nanoparticles and a higher phenolic content was recorded for CuMBGNs-50TA (170 ± 17 mg/mL) in comparison to ZnMBGNs-50TA (164 ± 18 mg/mL). However, there is no significant difference between the phenolic contents of CuMBGNs-50TA and ZnMBGNs-50TA (Fig. 4c). Sahiner produced poly(TA) using an oxidizing agent (sodium periodate) and reported that poly(TA) possesses total phenolic content of 202.0 ± 2.0 GAE μg/mL as compared to 168.0 ± 1.0 GAE μg/mL of TA [47]. In a similar study by Damian-Buda et al., clove oil was loaded into amino-functionalized MBGNs and the authors demonstrated that increasing the content of clove oil led to increasing the total phenolic content from 38 ± 1 GAE μg/mL to 56 ± 3 GAE μg/mL [48]. In another study, Bider et al. fabricated alginate dialdehyde-gelatin hydrogels containing various concentrations of ferulic acid and the total phenolic content was found to increase with increasing ferulic acid content [49].

As mentioned earlier, reactive free radicals can be efficiently quenched by phenolic compounds. As a natural polyphenol, TA is known to act as an appropriate antioxidant *via* the mechanism of hydrogen atom transferring from a phenolic group [50]. To investigate the free radical scavenging activity of antioxidants, DPPH with absorption band at 517 nm is usually utilized [51,52]. As a stable free radical, DPPH can accept an hydrogen radical or an electron, leading to formation of DPPH–H (non-radical form). This radical reduction happens *via* antioxidant substances’ hydrogen atoms, leading to a decrease in DPPH absorption band at 517 nm [[51], [52], [53]]. Therefore, in this study, the free radical scavenging efficiency of the TA-loaded nanoparticles was investigated using the DPPH assay. Fig. 4d illustrates the DPPH radical scavenging percentage of CuMBGNs and ZnMBGNs before and after loading with TA. The TA loading endowed CuMBGNs and ZnMBGNs with antioxidant activity. The DPPH free radical scavenging activity of the TA-loaded nanoparticles was increased with increasing concentration of TA (from 3 to 5 mg/mL) and the highest DPPH free radical scavenging activity was exhibited by CuMBGNs-50TA (88 ± 2 %) as compared to other samples. These findings are in agreement with the results of the FC assay, where CuMBGNs and ZnMBGNs with a higher amount of TA showed higher phenolic content, and therefore higher antioxidant activity. In a study by Wang et al., BG nanoparticles were coated with ε-poly-L-lysine and poly TA to accelerate hemostasis and wound healing [50]. The authors confirmed that the nanoparticles possessed free radical scavenging activity (more than 80 %), which was attributed to poly TA coating [50]. Li et al., fabricated antioxidant Cu-poly TA nanoparticles for the treatment of infected wounds. At the concentration of 50 μg mL^−1^, the nanoparticles led to 80.29 % of DPPH radicals elimination [54]. Wei et al. designed a series of physical and chemical cross-linked hydrogels composed of alginate, TA and gelatin methacrylate for wound healing applications. As results demonstrated, antimicrobial and antioxidant properties of the hydrogels were improved with increasing content of TA [55]. Our present study also confirmed that TA loading imparted free radical scavenging activity to CuMBGNs and ZnMBGNs. However, further investigation is needed in future to evaluate in detail the antioxidant mechanism of TA.

### 3.4 Effect of TA-loaded nanoparticles on hemolysis and hemostasis

**Fig. 5:**
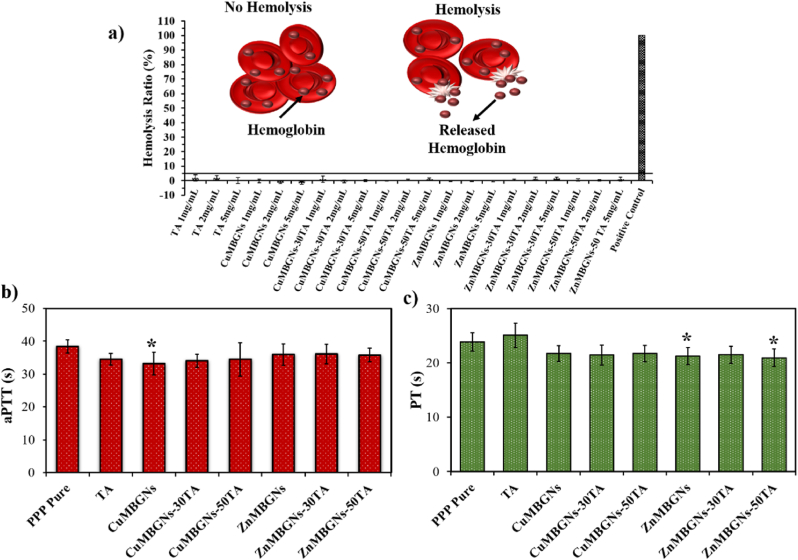
a) Hemolysis ratios (%) of TA, ZnMBGNs and CuMBGNs before and after loading. The solid line specifies the hemolysis ratio of 5 %. (b) aPTT and (c) PT of PPP after incubation with 1 mg/mL nanoparticles. Data were analyzed using one-way ANOVA (Tukey Test). Error bars show the mean ± standard deviation. The experiments were performed using blood from three donors and were performed in triplicate for each sample. The significant differences (p < 0.05) are marked by (∗) as compared to PPP pure.

As the biomaterials developed for hemostasis and wound healing applications can make direct contact with blood during their application at the wound site, they may lead to the breaking down and rupturing of red blood cells (erythrocytes), thereby provoking the release of erythrocytes’ components (hemoglobin) into the surrounding fluid, which is referred to as “hemolysis”. Therefore, one of the preliminary assessments of biomaterials for wound healing application is related to their hemolytic (hemolysis ratio: >5 %) or non-hemolytic (hemolysis ratio: < 5 %) characteristics [56,57]. In our study, the hemocompatibility of the TA-loaded ZnMBGNs and CuMBGNs was investigated at three different concentrations, namely 1, 2 and 5 mg/mL, and the results are shown in Fig. 5a. No hemolytic effect was observed for pure TA, loaded and unloaded nanoparticles as all groups demonstrated a hemolysis ratio of <5 %, even at the high concentrations. These results confirmed that TA-loaded ZnMBGNs and TA-loaded CuMBGNs are compatible towards erythrocytes.

To discover the impact of TA-loading on the blood coagulation cascade, PT (representing exogenous coagulation time) and aPTT (representing endogenous coagulation time) were measured using blood from healthy donors [58]. As it can be seen from Fig. 5b, in concentration of 1 mg/mL, the aPTT values of pure TA (∼34.47 s) and TA-loaded CuMBGNs (∼34–∼34.46 s) were lower in comparison to PPP pure (∼38.42 s) and the TA-loaded ZnMBGNs (∼35.77–∼36 s). However, the PT values (Fig. 5c) of the TA-loaded nanoparticles were in an almost similar range (∼20.93–∼21.73 s) and lower than PPP pure (23.88 s). Notably, the PT value increased when PPP was in contact with pure TA (∼25 s). While there are some reports that TA can promote a coagulation cascade, in a study by Deng et al., it was reported that with increasing concentration of TA (from 0.0001 to 1 mg/mL), both endogenous and exogenous coagulation pathways could be prolonged [58]. However, in our study the negative effect of TA on the exogenous coagulation pathway was reduced when TA was coated on both the ZnMBGNs and CuMBGNs. A parameter affecting the blood coagulation cascade that can be taken into account, is the presence of Ca^2+^ ions in the composition of TA-loaded nanoparticles. When the ions are released from the TA-loaded MBGNs, they could contribute to activating the blood coagulation cascade.

### 3.5 Cytocompatibility with NHDF cells

**Fig. 6:**
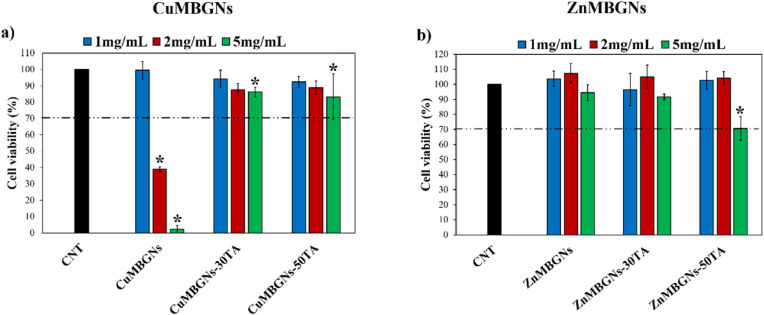
NHDF cell viability after treatment with the extracts of a) CuMBGNs and TA-loaded CuMBGNs; and b) ZnMBGNs and TA-loaded ZnMBGNs for 48 h at different concentrations: 1 mg/mL, 2 mg/mL and 5 mg/mL. The dashed line specifies the level of cell viability of a biocompatible material (70 %) according to international standard. Data were analyzed using one-way ANOVA (Tukey Test). Error bars illustrate the mean ± standard deviation (samples in triplicates). The significance differences marked by (∗) are displayed in comparison to the control.

Cytotoxicity is one of the crucial parameters that needs to be assessed before testing biomaterials in *in vivo* studies and clinical trials. The viability of NHDF cells was therefore assessed after contacting with the releasing products of the unloaded and loaded nanoparticles for 48 h at different concentrations (1, 2 and 5 mg/mL). After contacting the cells with the extract of CuMBGNs which was prepared *via* incubation of the nanoparticles in DMEM for 24 h, increasing the nanoparticle concentration (from 1 to 5 mg/mL) resulted in a strong reduction of NHDF cell viability, while the presence of TA reduced the cytotoxic behavior of CuMBGNs on NHDF cells (Fig. 6a). In our prior research, ion release analysis showed that at the concentration of 1 mg/mL, 2CuMBGNs, with the capability to release ∼15 mg/L Cu after 1 day incubation in DMEM, are biocompatible (more than 90 % NHDF cell viability) [29]. We assume that the cytotoxicity of CuMBGNs at higher concentrations may be attributed to the higher concentration of released Cu ions. Despite the beneficial effect of Cu ions, they have been known to lead to cytotoxicity at high concentrations. As reported, the toxicity of Cu may be ascribed to its redox properties. Cu ions can exist in an oxidized state (Cu^2+^) or a reduced state (Cu^1+^). During the transition between these two states, hydroxyl radicals (a type of ROS) can be produced, which is detrimental to DNA, proteins and lipids, thereby damaging heathy cells [24,[59], [60], [61]]. Paterson et al. found that while Cu-MBG nanoparticles were not toxic to human primary fibroblast cells at a concentration of 0.1 mg/mL, the cellular metabolic activity was reduced by Cu-MBG nanoparticles at a concentration of 1 mg/mL [26]. The lower cytotoxicity of TA-loaded CuMBGNs as compared to CuMBGNs may be attributed to the ability of TA to complex with Cu^2+^ which may interrupt Cu ion release from the nanoparticles, and therefore reduce the concentration of Cu released into the biological environment, thus decreasing Cu toxic effect. Additionally, TA-loaded and unloaded ZnMBGNs showed cytocompatibility when they came into contact with NHDF cells, except ZnMBGNs-50TA, which decreased NHDF viability to less than 80 % at the concentration of 5 mg/mL (Fig. 6b). Our results are consistent with previous studies, where TA or poly-TA-loaded nanoparticles were found to be cytocompatible towards a variety of cells, including C2C12, L929, RAW 264.7 and HUVECs cells [21,50]. It may be speculated that the release of both TA and Zn into the medium, in addition to the medium condition (e.g.*,* pH) and used concentrations, contributed synergistically to the reduction of theviability of NHDF cells when they were treated with ionic dissolution products of ZnMBGNs-50TA. We assume that increasing the concentration of TA loading and the concentration of ZnMBGNs-50TA to 5 mg/mL, led to the release of more ions and TA molecules, which were poorly attached to the ZnMBGNs. When exposed to the medium containing NHDF cells, this release reduced the viability of the NHDF cells.

In contrast to Cu, Zn is stable as Zn^2+^ and is generally reported to be redox inert; therefore, does not readily participate in redox reactions. However, it is also reported that cytotoxic effects in cells can be triggered by increasing the concentration of Zn so that stress in mitochondrial and cellular environments can be induced, thereby affecting the mitochondrial structure and resulting in cytotoxicity. Additionally, oxidative stress levels are reported to escalate by excessive concentrations of Zn [30,62,63]. In a study by Zhang et al., magnesium chloride (MgCl_2_) and TA were incorporated into bacterial cellulose (BC), leading to the formation of BC-TA-Mg composites. The result of this study showed that the L929 fibroblast cell viability decreased when the cells were treated with BC-TA, as compared to BC-TA-Mg composites. The authors attributed the reduced cell viability to the higher concentration of TA which was freely released to the medium. However, they believed that in presence of Mg^2+^^,^ which could interact with TA, less TA could be released into the medium, thereby improving the biocompatibility [64]. In another study by Neščáková et al., mouse embryonic fibroblast cell viability decreased when the cells were treated with elution extracts from Zn-MBGNs at concentration of 5 mg/L, as compared to dissolution products of Zn-free MBGNs, which did not cause any negative effect on the cell viability at the same concentration [31]. NHDF cell death resulting from apoptosis after treatment by ZnO NPs was also reported by Meyer et al. The results of this study demonstrated that increasing concentration of ZnO NPs from 2.5 to 100 μg/mL reduced NHDF cell viability. It was found that apoptosis was induced in NHDF cells by ZnO NPs *via* the upregulation of two proteins, namely p53 and phospho-p38 [65]. In our previous work, ion release studies in DMEM showed that the concentrations of Cu released from 2CuMBGNs was around 15 mg/L, which was higher than the Zn concentration released from 2ZnMBGNs, which was around 6 mg/L after 24 h incubation [29]. In this study, the toxicity of ZnMBGNs was found to be less affected by increasing the concentration, which may be ascribed to the slower and lower release of Zn in comparison to the rapid and higher release of Cu from CuMBGNs. Overall, these results verified that TA loading did not negatively affect NHDF cell viability at least at low concentrations. However, future research is needed to clarify all the contributing factors to cytotoxicity of MBGNs-TA at higher concentrations.

**Fig. 7:**
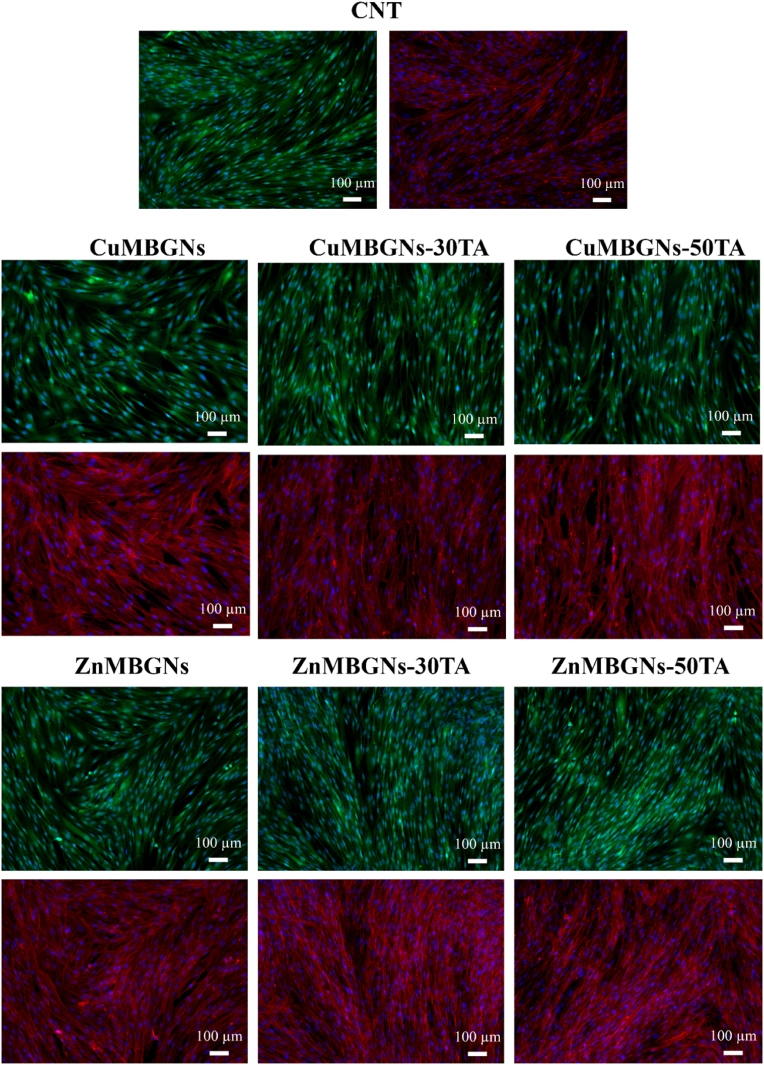
Fluorescence microscopy images showing NHDF cells stained with DAPI (blue), Calcein AM (green) and rhodamine-phalloidin (red) after 48 h treatment with dissolution products of ZnMBGNs, ZnMBGNs-TA, CuMBGNs and CuMBGNs-TA at a concentration of 1 mg/mL (Scale bar = 100 μm).

Fluorescence microscopy was used to observe the morphology of cells after contacting with the extracts of TA-loaded nanoparticles at a concentration of 1 mg/mL (Fig. 7). The actin filament, cytoplasm and nucleus of living cells were stained with rhodamine-phalloidin (red), Calcein AM (green) and DAPI (blue), respectively. The rhodamine-phalloidin/DAPI results showed cytoplasmic filamentous distributions (red-colored) of F-actin with a prominent nucleus (blue) for NHDF cells. Moreover, it can be observed that the NHDF cells had spindle-shaped morphology, indicating that cells have no morphological abnormality.

### 3.6 Wound scratch assay

**Fig. 8:**
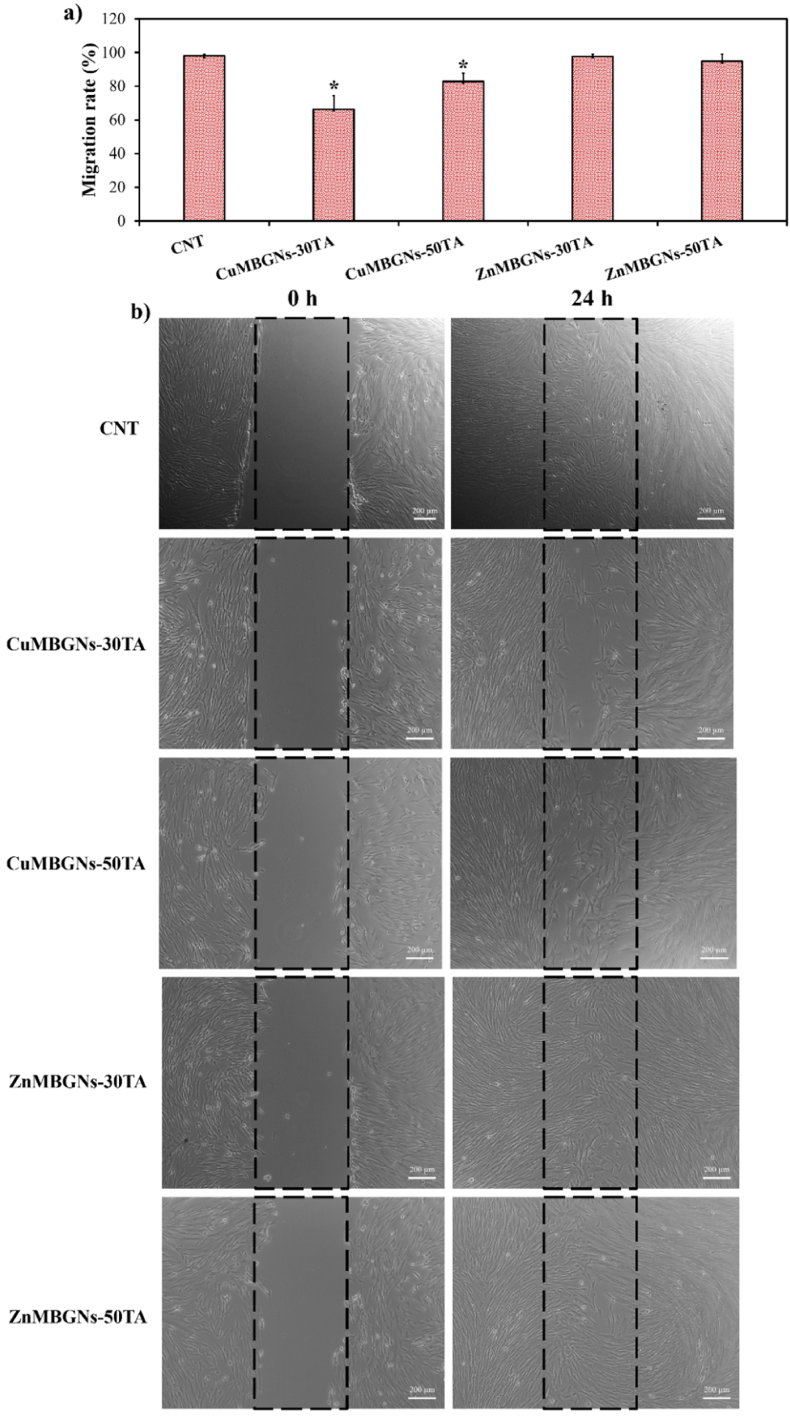
a) Wound closure ratios of NHDF cells after exposure to leaching products of CuMBGNs-TA and ZnMBGNs-TA at a concentration of 1 mg/mL. Data were analyzed using one-way ANOVA (Tukey Test). Error bars display the mean ± standard deviation (samples in four replicates). The significance differences marked by (∗) are demonstrated as compared to the control; b) microscopy images of the scratch areas at time 0 h and after 24 h treatment.

Dermal fibroblast cells are important during wound healing and their migration and proliferation are vital in granulation tissue formation [66]. In this study, the effect of TA-loaded nanoparticles on the migration behavior of NHDF cells was evaluated *via* the *in vitro* scratch assay. The wound closure rates (Fig. 8a) and microscopy images (Fig. 8b) of the scratch areas after 0 and 24 h exposure to the dissolution products of TA-loaded CuMBGNs and ZnMBGNs, which were obtained *via* incubation of the particles (1 mg/mL) in DMEM for 24 h, are depicted in Fig. 8. As the results demonstrated, NHDF cell migration was observed with ZnMBGNs-TA and CuMBGNs-TA leachates after 24 h treatment. While the wound closure rates were lower for CuMBGNs-TA leachates (∼66 %–∼83 %) as compared to the control (∼98 %), NHDF cells migrated to fill the scratch area with ∼95 % – ∼98 % of wound closure rates when they were exposed to ZnMBGNs-TA leachates, similar to the control. As previously proven, Cu plays an important role in all stages of wound healing [67,68]. It has been reported that not only the proliferation and migration of keratinocytes and fibroblasts can be promoted by Cu ions, but also it essentially contributes to the regulation of growth factors such as angiogenin and vascular endothelial growth factor (VEGF), thereby accelerating angiogenesis [61,67,[69], [70], [71]]. The role of Cu in collagen production and in the formation of elastin fibers by dermal fibroblast has been also reported, which plays a vital role for tissue formation during the phases of proliferation and remodeling in the wound healing process [71]. It should be however stated that the effect of Cu ions on the function of cells is dose-dependent. In our study, the amount of Cu ions present in CuMBGNs-TA leachates probably negatively affected the NHDF cell migration ability. It is also presumed that many other factors can affect the cell migration, such as the release of other ions (e.g., Si and Ca), the release of poorly attached TA molecules, and the pH of the medium. In a study by Alizadeh et al., CuNPs in various concentrations and sizes were developed and assessed for wound healing application. The study reported that HDF cell migration increased in size and concentration dependent manners so that HDF cells migrated faster when the cells were treated with CuNPs in size of 20 nm and 1 μM concentration as well as in the size of 40 nm and concentrations of 10 μM and 100 μM, as compared to other concentrations and sizes. Moreover, the *in vivo* results showed that would healing was promoted over shorter periods of time by CuNPs in the concentration and size of 1 μM and 80 nm, respectively, by granulation tissue production and higher blood vessel formation [72].

In our study, ZnMBGNs-TA did not show adverse effects on the migration ability of NHDF cells at a concentration of 1 mg/mL. Human skin contains a high amount of Zn and Zn-deficient conditions have been reported to slow down the wound healing process [73]. It is proven that Zn plays a critical role in the wound healing process, ranging from the blood clotting cascade, inflammation, epithelial regeneration, angiogenesis to scar production [73]. In a study by Yang et al., released Zn ions from magnesium/methacrylate gelatin/Zn hydrogel was found to promote migration of human immortalized keratinocytes (HaCats) and human skin fibroblast (HSCFs) [74]. As the transformation of fibroblasts into myofibroblasts can result in contraction, closing wounds and production of proteins and growth factors which plays a vital role in the wound healing process, the result of the study also suggested that the entry of Zn ions into HSCFs could promote differentiation of HSFs into myofibroblasts, thereby enhancing skin defect healing [74]. In another study by Mutlu et al., human keratinocyte migration was additionally improved by the dissolution products of chitosan-Zn films, which was ascribed to the presence of Zn ions [75].

## 4 Conclusions

In this study, TA-loaded CuMBGNs and ZnMBGNs were synthesized and evaluated *via* a series of physicochemical characterizations and biological experiments for wound healing application. The results revealed that TA was successfully loaded onto the nanoparticles by physical adsorption and was able to be continuously released in PBS over 28 days incubation. The loading of TA endowed CuMBGNs and ZnMBGNs with DPPH radical scavenging activity while preserving their cytocompatibility and hemocompatibility. However, the viability of NHDF cells decreased by increasing the nanoparticle concentration from 1 mg/mL to 5 mg/mL. TA loading decreased the cytotoxic effect of CuMBGNs at higher concentrations of 2 and 5 mg/mL, which may be the result of interactions between Cu ions and TA, thereby releasing less Cu ions. At a concentration of 1 mg/mL, the ZnMBGNs-TA leachates also resulted in higher *in vitro* wound closure rate as compared to the releasing products of CuMBGNs-TA, which may be assigned to the therapeutic effect of Zn ions. Based on the findings presented in this study, it can be stated that CuMBGNs and ZnMBGNs containing therapeutic ions (Si, Ca, Cu and Zn) are suitable candidates to carry and deliver TA molecules. To validate the full potential of TA-loaded CuMBGNs or ZnMBGNs for wound healing applications, more research needs to be conducted both *in vitro* and *in vivo*. Nevertheless, our findings open up new research avenues to explore the combined advantages of polyphenols and therapeutic ion-containing MBGNs with antioxidant properties for wound healing applications.

## CRediT authorship contribution statement

**Sara Pourshahrestani:** Writing – original draft, Visualization, Validation, Methodology, Investigation, Funding acquisition, Formal analysis, Data curation, Conceptualization. **Irem Unalan:** Writing – review & editing, Methodology, Investigation. **Ehsan Zeimaran:** Writing – review & editing, Investigation, Formal analysis. **Zhiyan Xu:** Writing – review & editing, Investigation. **Judith A. Roether:** Writing – review & editing, Investigation. **Andrea Kerpes:** Writing – review & editing, Methodology, Investigation. **Christina Janko:** Writing – review & editing, Resources, Methodology, Investigation, Funding acquisition. **Christoph Alexiou:** Writing – review & editing, Resources. **Aldo R. Boccaccini:** Writing – review & editing, Supervision, Resources, Project administration, Funding acquisition, Conceptualization.

## Data availability

The data are available from the corresponding authors upon reasonable request.

## Ethics approval and consent to participate

Blood was collected from human healthy volunteers and the use of human blood was approved by the ethics committee of the University of Erlangen–Nuremberg (# 21-383-2-B). Appropriate consents have been obtained from the volunteers.

## Declaration of competing interest

Prof. Aldo R. Boccaccini is an Associate Editor for Bioactive Materials and was not involved in the editorial review or the decision to publish this article. All authors declare that there are no competing interests.
